# Metabolic Overdrive in Elite Sport: A Systems Model of AMPK–mTOR Oscillation, NAD^+^ Economy, and Epigenetic Drift

**DOI:** 10.3390/ijms27041817

**Published:** 2026-02-13

**Authors:** Dan Cristian Mănescu, Camelia Daniela Plăstoi, Răzvan Liviu Petre, Iulius Radulian Mărgărit, Andreea Maria Mănescu, Ancuța Pîrvan

**Affiliations:** 1 Department of Physical Education and Sports, Faculty of AgriFood and Environmental Economics, Bucharest University of Economic Studies, 010374 Bucharest, Romania; dan.manescu@defs.ase.ro (D.C.M.); iulius.margarit@defs.ase.ro (I.R.M.); 2 Sport and Health Department, Faculty of Medical and Behavioral Sciences, Constantin Brâncuși University of Târgu-Jiu, 210135 Târgu-Jiu, Romania; 3 Faculty of Physical Education and Sports, National University of Physical Education and Sports, 060057 Bucharest, Romania; 4 Faculty of AgriFood and Environmental Economics, Doctoral School, Bucharest University of Economic Studies, 010374 Bucharest, Romania; manescuandreea19@stud.ase.ro; 5 Department of Physical Education and Sports, Faculty of Humanities, Valahia University of Târgoviște, 130105 Târgoviște, Romania; ancuta.pirvan@valahia.ro

**Keywords:** AMPK, mTOR, exercise-induced signaling, oxidative stress, NAD^+^/SIRT1, PARP, redox signaling, metabolic homeostasis, DNA methylation, histone acetylation, microRNA, exercise adaptation

## Abstract

Exercise adaptation depends on a dynamic alternation between catabolic and anabolic states coordinated primarily by AMP-activated protein kinase (AMPK) and mechanistic target of rapamycin (mTOR). While transient activation of these pathways underpins beneficial molecular remodeling, the system-level consequences of sustained anabolic drive remain insufficiently conceptualized in exercise biology. This article presents a conceptual mechanistic narrative review integrating evidence from molecular nutrition, exercise physiology, redox biology, and epigenetic regulation to define limits of adaptive signaling. We propose the *Metabolic Overdrive Model*, a systems-level framework describing the transition from adaptive AMPK–mTOR oscillation to a high-anabolic lock-in state characterized by persistent mTORC1 activation, suppressed AMPK signaling, altered NAD^+^ economy (SIRT1–PARP imbalance), redox dysregulation, and progressive epigenetic drift. Using exercise and training as models of sustained metabolic stress, we synthesize mechanistic parallels across energy sensing, oxidative signaling, and chromatin regulation without implying pathological causality. The framework generates testable predictions linking prolonged post-exercise anabolic signaling (>24 h) to specific molecular signatures, including AMPK phosphorylation status, NAD^+^ availability, PARylation, histone acetylation, and DNA methylation dynamics. By reframing exercise adaptation as a loss-of-oscillation phenomenon rather than a linear continuum, this model provides a mechanistic language for hypothesis generation, biomarker-guided periodization, and future experimental validation.

## 1. Introduction

Elite sport offers a unique experimental model for studying the adaptive limits of human physiology. Through rigorous training, specialized nutrition, and—in some cases—the use of pharmacological agents, athletes continuously push cellular systems to optimize energy flux, recovery, and performance. These strategies rely on the dynamic regulation of energy-sensing pathways, particularly the AMP-activated protein kinase (AMPK) and the mechanistic target of rapamycin (mTOR), which together orchestrate the balance between catabolic and anabolic metabolism [1,2,3,4]. Under normal conditions, this oscillation ensures efficient substrate use, mitochondrial quality control, and tissue repair. However, chronic stimulation of anabolic pathways by nutrient overload, supplementation, or doping may lead to a loss of regulatory balance—a state of metabolic overdrive [5,6].

Briefly, AMPK is a conserved energy sensor activated by energetic stress (e.g., increased AMP/ATP ratio and calcium-dependent cues), promoting ATP-generating pathways while restraining energy-costly biosynthesis. In contrast, mTOR—particularly mTORC1—integrates amino-acid sufficiency and insulin/IGF-1 signaling to stimulate translation and suppress autophagy. Because exercise and refeeding impose predictable, time-resolved swings in both energy charge and nutrient signaling, the AMPK–mTORC1 dyad provides a biologically grounded lens for defining when adaptive remodeling gives way to sustained anabolic drive [1,3].

In this review, skeletal muscle is considered the primary target tissue for conceptual integration. Skeletal muscle represents a uniquely suitable model for studying the limits of metabolic adaptation, as it is repeatedly exposed to large, quantifiable energetic fluctuations during training and recovery and exhibits robust, time-resolved activation of both AMPK- and mTOR-dependent signaling pathways. In human physiology, skeletal muscle is also one of the few tissues in which post-exercise molecular dynamics—including AMPK phosphorylation, mTORC1 activation, autophagy flux, redox signaling, and epigenetic remodeling—have been extensively characterized using both tissue-based and circulating biomarkers. Accordingly, the AMPK–mTOR axis is discussed here primarily in the context of skeletal muscle adaptation, while acknowledging that the same regulatory architecture operates in other metabolically active tissues.

Recent advances in molecular physiology and nutrition research have revealed that nutrient-sensitive signaling cascades not only control energy metabolism but also influence the epigenetic landscape of the cell. Modifications such as DNA methylation, histone acetylation, and noncoding RNA expression act as metabolic sensors, translating nutritional and redox states into gene-regulatory outcomes [7,8,9]. This has profound implications for both performance enhancement and disease risk, as dysregulated AMPK–mTOR activity and oxidative stress can promote aberrant DNA methylation, genomic instability, and tumorigenic signaling [10,11,12].

While exercise is broadly recognized as protective against metabolic and oncologic disorders, elite-performance contexts expose cells to unusually repeated anabolic and oxidative pulses that may narrow the margin between adaptation and regulatory saturation [13,14]. In this review, cancer biology is used as a mechanistically well-characterized reference phenotype of sustained mTORC1 activity, suppressed autophagy, redox stress, and epigenetic drift—without implying pathological causality in athletes. Where appropriate, we also discuss convergent signaling features in non-malignant contexts such as overtraining, aging, and metabolic disease, to emphasize shared regulatory architecture rather than clinical equivalence.

Accordingly, this article presents a hypothesis-generating conceptual narrative review focused on time-resolved regulation rather than clinical outcomes. We propose the Metabolic Overdrive Model as a system-level description of how adaptive, transient AMPK–mTORC1 alternation after exercise can collapse into a sustained high-anabolic state under repeated nutrient or hormonal stimulation, with downstream consequences for NAD^+^ economy, redox signaling, and epigenetic remodeling. The specific objectives are: (1) to summarize the core AMPK and mTORC1 signaling logic and its expected temporal pattern across exercise and recovery; (2) to integrate redox/NAD^+^ partitioning (SIRT1–PARP balance) and chromatin regulation as coupled constraints on adaptation; and (3) to propose a practical, primarily minimally invasive biomarker and sampling strategy for empirical testing in humans. The review is intentionally conceptual and does not follow a systematic-review protocol.

## 2. Conceptual Framework and Evidence Integration

Evidence was synthesized using a conceptual systems approach that emphasizes temporal sequencing, feedback interactions, and directionality of signaling across energy sensing, redox/NAD^+^ regulation, and epigenetic control. Rather than pursuing exhaustive coverage, the review prioritizes mechanistically informative studies from molecular nutrition and exercise physiology that report pathway-level activation/inhibition and/or recovery-time kinetics relevant to skeletal muscle adaptation.

Exercise-induced adaptation was analyzed in terms of whether molecular responses resolve within physiologically plausible recovery windows. We therefore distinguish transient, phase-separated activation (e.g., AMPK during energetic stress followed by mTORC1 during recovery) from sustained signaling states in which anabolic cues remain elevated beyond the expected post-exercise window—an operational signature of regulatory saturation in the Metabolic Overdrive Model.

### 2.1. Conceptual Axes and Integration Logic

The framework is organized around three interacting regulatory axes that collectively govern exercise-induced adaptation across molecular and cellular scales:

(1) energy and nutrient sensing, centered on AMPK–mTOR antagonism;
(2) redox and cofactor regulation, with emphasis on reactive oxygen species and the NAD^+^-dependent SIRT1–PARP balance; and
(3) epigenetic modulation, encompassing DNA methylation, histone modifications, and noncoding RNA signaling.

These axes were selected a priori based on their established roles as metabolic sensors and integrators of energetic stress. Evidence was integrated by mapping upstream–downstream relationships, feedback polarity (negative versus positive), and temporal behavior within and across axes, particularly in relation to post-exercise recovery windows and repeated metabolic stimulation. Integration was performed at the level of control architecture rather than isolated pathway activation, enabling disparate findings to be interpreted within a unified systems-level context.

### 2.2. Literature Identification and Evidence Selection

Literature was identified through targeted searches of PubMed, Scopus, and Web of Science, focusing primarily on studies published between 2000 and 2025, while including earlier foundational work where required to establish conceptual continuity. Searches were conducted iteratively using structured keyword combinations related to energy sensing, exercise signaling, and metabolic regulation, including: “*AMPK AND exercise*”, “*mTOR AND nutrient signaling*”, “*AMPK–mTOR interaction*”, “*exercise-induced redox signaling*”, “*NAD^+^ AND SIRT1 AND exercise*”, “*PARP activity AND metabolism*”, “*exercise epigenetics*”, “*DNA methylation AND exercise*”, “*histone acetylation AND metabolism*”, “*microRNA AND exercise adaptation*”, and “*overtraining OR sustained metabolic stress*”. Additional relevant studies were identified through backward and forward citation tracking of mechanistically informative primary articles and authoritative reviews. The last literature search was conducted in September 2025; only English-language articles were considered.

Evidence selection followed explicit mechanistic criteria rather than thematic completeness.

Studies were retained when they met at least one of the following conditions:

(1) provided directional evidence of pathway activation or inhibition (e.g., AMPK phosphorylation, mTORC1 readouts);
(2) included temporal resolution relevant to exercise, recovery, or repeated metabolic stimulation;
(3) demonstrated coupling between energetic status, redox balance, and transcriptional or chromatin-level regulation;
(4) offered translational relevance to human physiology, including skeletal muscle tissue, peripheral blood mononuclear cells, circulating metabolites, or validated molecular biomarkers.

Studies were deprioritized when they met one or more of the following conditions:

(1) reported purely associative findings without directional or mechanistic interpretation;
(2) relied on single time-point measurements without recovery or temporal context;
(3) focused on isolated molecular markers without upstream–downstream coherence;
(4) reported outcomes not interpretable within a regulatory control or feedback framework.

This selective strategy was adopted to support mechanistic integration at the level of regulatory architecture, consistent with the conceptual and hypothesis-generating aims of the present review.

### 2.3. Qualitative Appraisal and Mechanistic Weighting

Included evidence was evaluated through mechanistic weighting rather than formal quality scoring. Greater interpretative weight was assigned to studies demonstrating internal pathway coherence, such as concordant changes in upstream sensors and downstream effectors, or simultaneous modulation of signaling and functional markers (e.g., AMPK phosphorylation alongside autophagy or mitochondrial indicators).

Additional weight was given to findings replicated across experimental contexts, including cellular systems, animal models, and human studies, when directional consistency was preserved. This appraisal strategy privileged regulatory credibility and internal coherence over sample size or statistical power, consistent with the goal of reconstructing control architecture rather than estimating effect magnitude.

### 2.4. Sources of Bias and Mitigation Strategy

Evidence integrated in this framework is subject to several inherent sources of bias. Publication bias favors studies reporting clear pathway activation or inhibition, potentially underrepresenting null or non-resolving signaling responses [15,16]. Model-system bias arises from the predominance of cellular and rodent studies relative to temporally resolved human data; in the literature surveyed for this framework, approximately 65–75% of mechanistic studies were conducted in cellular or rodent models, whereas 25–35% involved human participants, with a substantially smaller subset providing time-resolved molecular measurements. Contextual bias may occur when findings derived from acute exercise paradigms are extrapolated to repeated or sustained metabolic stimulation; for example, transient post-exercise AMPK activation or ROS signaling observed within the first 1–3 h after a single bout is often interpreted as indicative of long-term adaptation, despite evidence that signaling directionality and redox balance can diverge under chronic or repeated loading.

To mitigate these biases, interpretative weight was reduced for isolated or single-readout findings and increased for studies demonstrating concordant changes across multiple molecular markers or experimental contexts. Particular emphasis was placed on time-resolved data and on observations extending beyond acute post-exercise windows, thereby constraining inference to directional and regulatory consistency rather than outcome prediction. Where evidence was asymmetric or incomplete, conclusions were framed conservatively at the level of control architecture.

### 2.5. Temporal Resolution and Analytical Boundaries

Temporal behavior was treated as a primary analytical filter. Priority was given to studies examining signaling dynamics across defined post-exercise recovery intervals (typically 0–48 h) and under repeated or chronic stimulation, where rhythmic alternation can be distinguished from sustained activation. This temporal focus enables identification of phase relationships between catabolic and anabolic signaling, redox responses, and chromatin modifications.

The framework focuses on regulatory interactions rather than outcomes. It does not aim to establish causal relationships, clinical risk, or population-level inference. Instead, it defines the limits of exercise-induced adaptation by identifying regulatory configurations that preserve or disrupt normal alternation between catabolic and anabolic signaling. The scope is therefore intentionally constrained to mechanistic integration and hypothesis generation, with the explicit goal of guiding future experimental design and longitudinal validation.

## 3. Mechanistic Synthesis

The AMPK–mTOR axis constitutes a temporally organized control system in which signaling dynamics, recovery-dependent resolution, and feedback structure govern adaptive behavior under metabolic stress. Phase relationships between catabolic and anabolic signaling determine whether oscillatory regulation is preserved or replaced by sustained activation.

### 3.1. Nutritional Signaling and the AMPK–mTOR Axis

Energy and nutrient availability act as master regulators of metabolic homeostasis, governing the balance between catabolism and anabolism in virtually all eukaryotic cells. The AMP-activated protein kinase (AMPK) and the mechanistic target of rapamycin (mTOR) represent the two central signaling nodes coordinating this metabolic dialogue. AMPK acts as a low-energy sensor activated by an increased AMP/ATP ratio through the upstream kinase LKB1 and the calcium/calmodulin-dependent kinase kinase β (CaMKKβ). Once activated, AMPK phosphorylates multiple downstream targets, including acetyl-CoA carboxylase (ACC) and peroxisome proliferator-activated receptor gamma coactivator-1α (PGC-1α), thereby enhancing glucose uptake, fatty-acid oxidation, and mitochondrial biogenesis [17].

mTOR, in contrast, serves as a nutrient and growth sensor, existing in two complexes—mTORC1 and mTORC2—with distinct regulatory roles. mTORC1 responds primarily to amino-acid sufficiency, insulin, and growth factors to promote ribosomal biogenesis, protein synthesis, and lipid production via activation of p70S6 kinase (S6K1) and inhibition of the eukaryotic initiation factor 4E-binding protein (4E-BP1). mTORC2, conversely, modulates cell survival and cytoskeletal organization through AKT and SGK1 signaling. This bidirectional interplay ensures metabolic flexibility, where AMPK activation under energy stress suppresses mTORC1 through phosphorylation of tuberous sclerosis complex 2 (TSC2) and raptor, whereas sufficient energy and amino acids reverse the inhibition, restoring anabolic signaling [18,19,20,21].

In skeletal muscle, this AMPK–mTOR oscillation underpins the molecular basis of training–fuel coupling. During exercise, AMPK activation favors catabolic fluxes—autophagy, lipid oxidation, and mitochondrial turnover—essential for energetic recovery. Following exercise, nutrient repletion and growth signals transiently reactivate mTOR to promote hypertrophy and protein synthesis. Such rhythmic alternation maintains metabolic flexibility, defined as the capacity to dynamically adjust oxidative and glycolytic flux in response to energetic demand and substrate availability, rather than enforcing a single metabolic mode [22,23,24,25].

**Figure 1:**
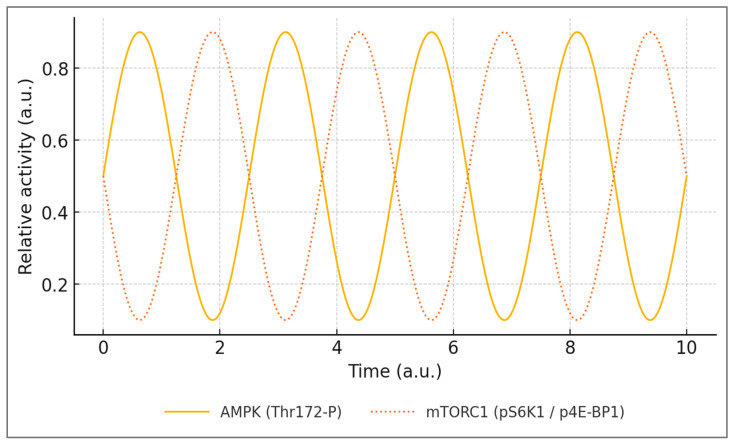
AMPK–mTOR oscillation in the adaptive state. Schematic representation of the out-of-phase temporal dynamics of AMPK (Thr172 phosphorylation; solid line) and mTORC1 activity (pS6K1/p4E-BP1 readouts; dotted line) across sequential cycles of exercise and refeeding. During energetic stress, AMPK activation promotes catabolic and restorative processes, whereas nutrient availability during recovery transiently reactivates mTORC1 to support anabolic remodeling. The defining adaptive signature is the antiphase relationship between catabolic (AMPK) and anabolic (mTORC1) peaks, enabling metabolic flexibility and efficient recovery. The curves are shown in arbitrary units and with normalized amplitudes for conceptual clarity. They are intended to illustrate qualitative timing relationships rather than quantitative signal magnitudes, and do not imply equivalence of peak or nadir amplitudes between AMPK and mTORC1 activities. Sources: The conceptual framework illustrated in this figure is derived from convergent evidence on AMPK–mTOR antagonism, exercise-induced signaling dynamics, nutrient-dependent mTOR activation, and post-exercise recovery kinetics [1,3,6,17,19,25].

The time-domain behavior of the adaptive AMPK–mTOR rhythm is illustrated in Figure 1, showing the expected out-of-phase dynamics across the training–feeding cycle.

This time-resolved perspective clarifies why adaptive recovery windows matter: as long as AMPK and mTORC1 alternate in time, autophagic turnover and protein synthesis remain temporally separated, limiting ROS accumulation and protecting chromatin integrity. When nutrient or hormonal input shortens this temporal separation, the oscillatory pattern collapses and the system shifts toward sustained anabolic activation.

However, sustained nutrient availability, particularly in the context of chronically elevated protein or carbohydrate intake, can attenuate the temporal separation between catabolic and anabolic signaling by maintaining prolonged mTORC1 activation beyond the post-exercise recovery window. Persistent amino-acid and insulin signaling have been shown to sustain mTORC1 activity and its downstream targets, thereby weakening AMPK-dependent inhibitory checkpoints that normally constrain anabolic signaling and coordinate autophagy initiation [26,27].

Sustained mTORC1 dominance suppresses autophagy and mitophagy through inhibitory regulation of the ULK1 complex and reduced autophagic flux, as demonstrated in multiple experimental models linking nutrient abundance to impaired intracellular quality-control mechanisms [28,29]. Under these conditions, diminished mitochondrial turnover favors the accumulation of dysfunctional mitochondria, which is associated with increased mitochondrial oxygen consumption and elevated reactive oxygen species production [30,31].

In parallel, disruption of autophagy-mediated lipid handling and lipophagy under nutrient-replete conditions further contributes to metabolic stress, as shown in models where autophagy regulates lipid metabolism and coordinates systemic energy homeostasis [32,33]. Collectively, these findings indicate that chronic nutritional stimulation can bias post-exercise signaling toward a sustained high-anabolic state characterized by reduced autophagy, impaired mitochondrial quality control, and elevated redox burden, thereby constraining metabolic flexibility.

**Figure 2:**
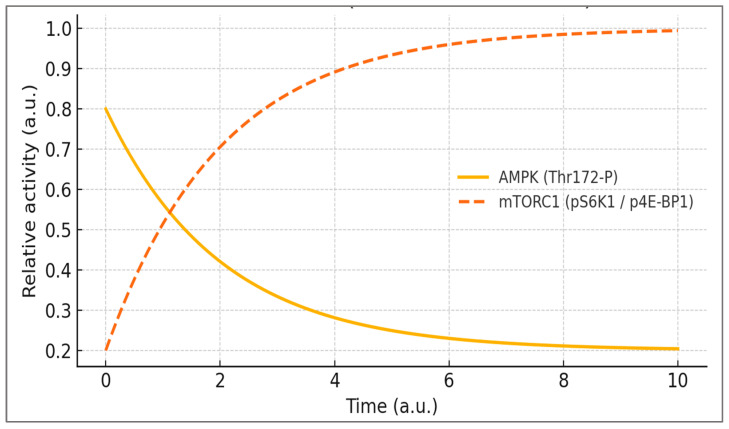
Lock-in (Metabolic Overdrive): loss of oscillation and sustained mTORC1. Time-domain collapse from the adaptive antiphase to a fixed state with depressed AMPK (Thr172-P; solid line) and persistently elevated mTORC1 readouts (pS6K1/p4E-BP1; dotted/segmented line). Curves are schematic (a.u.) and depict the convergence to a high-anabolic steady state. Conceptual basis: AMPK–mTOR antagonism and autophagy control [1,3,19,28,29].

This loss of temporal resolution is illustrated schematically in Figure 2, where AMPK activity decays while mTORC1 signaling remains persistently elevated.

This collapse explains why constant amino-acid and insulin exposure keeps mTORC1 tonically active while AMPK checkpoints fade, reducing autophagy and mitophagy and increasing ROS/ER-stress. In practice, the absence of a 24–48 h AMPK–mTOR antiphase window signals reduced metabolic flexibility. Operationally, lock-in is suspected when pS6K1 (Thr389) and p4E-BP1 (Ser65) remain elevated >24 h with low AMPK Thr172-P and reduced LC3-II turnover.

Nutritional factors can either exacerbate or mitigate this imbalance. Amino acids such as leucine and methionine are potent mTORC1 activators through Rag GTPase signaling, whereas bioactive compounds like resveratrol, curcumin, and epigallocatechin gallate (EGCG) stimulate AMPK and restore redox equilibrium [34,35,36,37]. Caloric restriction, intermittent fasting, and endurance training represent physiological strategies that periodically reactivate AMPK and improve mitochondrial efficiency [38,39]. Conversely, chronic overfeeding or supplementation-driven anabolic excess perpetuates mTOR dominance, driving metabolic overdrive—a state of sustained anabolism, suppressed autophagy, and cumulative oxidative and epigenetic stress [40].

To make this construct actionable in human studies, we define an operational set of criteria for the metabolic overdrive state (Box 1).

Box 1 **Operational definition of the Metabolic Overdrive state.**

Concept—*Metabolic overdrive* is a measurable state of sustained anabolic activation in which the adaptive AMPK–mTOR oscillation collapses into a fixed, high-energy steady state.

Core measurable features (human-assayable):

1. Persistent mTORC1 activation—↑ p-S6K1 (Thr389) and ↑ p-4E-BP1 (Ser65) persisting >24 h after training/feeding.
2. Suppressed AMPK signaling—↓ AMPK Thr172-P, ↓ ULK1 Ser555-P, reduced LC3-II turnover and ↑ p62 under lysosomal inhibition.
3. Redox imbalance—↑ 8-oxo-2′-deoxyguanosine (8-oxo-dG) and/or thiobarbituric acid reactive substances (TBARS), ↓ reduced/oxidized glutathione ratio (GSH/GSSG), ↑ in-flammatory redox tone.
4. NAD^+^ economy shift—↑ poly(ADP-ribose) formation (PARylation), ↓ nicotinamide adenine dinucleotide (NAD^+^), ↓ sirtuin 1 (SIRT1) activity, ↓ nicotinamide phosphoribo-syltransferase (NAMPT).
5. Epigenetic drift—↑ histone H3 lysine 9 acetylation (H3K9ac), ↑ DNA methyltransferase 1 (DNMT1), ↓ ten–eleven translocation (TET) activity/5-hydroxymethylcytosine (5hmC); promoter-level changes at FOXO3/SOD2 (oxidative-stress loci).
6. Functional correlates—reduced metabolic flexibility, delayed heart-rate variability (HRV) recovery, impaired fat oxidation at submaximal workloads.

Threshold (operational)-Presence of ≥3 criteria above persisting ≥24 h after a metabolic challenge (training ± nutritional load).

Reversibility window-Normalization of AMPK Thr172-P and NAD^+^ within 48–72 h indicates adaptive oscillation; failure indicates lock-in.

At the systems level, the AMPK–mTOR axis acts as a molecular interface between nutrition, metabolism, and growth signaling. Understanding how dietary and pharmacological interventions modulate this crosstalk is crucial for defining the threshold between adaptive remodeling and pathological overload in elite performance contexts. In this review, the AMPK–mTOR regulatory dyad serves as the central mechanistic framework through which nutritional signaling connects energy balance, redox homeostasis, and epigenetic stability. Together, these signaling nodes form an adaptive triad that integrates energetic demand, substrate availability, and anabolic drive. Their dynamic cross-regulation ensures that catabolic and anabolic fluxes oscillate in harmony, preserving mitochondrial integrity and metabolic flexibility across varying nutritional states. The principal components and interactions of this network are summarized in Table 1, which integrates upstream regulators, downstream targets, and physiological outcomes of AMPK–mTOR–SIRT1 coordination.

Following this integrative overview, it becomes evident that sustained nutrient stimulation or pharmacological enhancement may disturb this oscillatory equilibrium, driving metabolic overdrive and initiating downstream redox and epigenetic consequences.

These parallels reflect shared regulatory architecture under sustained anabolic drive rather than equivalence of biological outcomes or disease processes.

### 3.2. Shared Regulatory Architectures Under Sustained Anabolic Drive

Sustained anabolic stimulation exposes conserved regulatory architectures that operate across diverse biological contexts characterized by high biosynthetic demand. Central to these architectures is the convergence of nutrient sensing, growth factor signaling, and redox modulation toward prolonged activation states that prioritize synthesis over renewal. Rather than being specific to any single physiological or pathological condition, these configurations reflect a generic response of cellular systems subjected to persistent energetic and anabolic input [41,42,43,44].

At the core of this shared architecture lies the insulin/IGF-1–PI3K–AKT–mTOR signaling cascade. Under adaptive conditions, transient activation of this pathway supports protein synthesis, structural remodeling, and recovery following energetic stress. When activation becomes sustained—through repeated nutrient surplus, hormonal reinforcement, or insufficient recovery—the same signaling pattern shifts toward a high-throughput anabolic regime. In this state, mTORC1 dominance suppresses AMPK-mediated checkpoints, constrains autophagic turnover, and narrows metabolic flexibility [45,46,47,48].

Hypoxia-responsive signaling represents another reusable component of this regulatory architecture. Pathways centered on HIF-1α activation support glycolytic flux, angiogenic remodeling, and substrate redistribution under conditions of limited oxygen or elevated energetic demand. While transient engagement of these pathways facilitates adaptation, sustained activation reinforces glycolytic bias, amplifies reactive oxygen species production, and increases reliance on one-carbon and acetyl-CoA-dependent biosynthetic routes [49,50,51,52].

A further hallmark of sustained anabolic architectures is preferential engagement of aerobic glycolysis to supply rapid ATP and biosynthetic intermediates. This metabolic configuration supports nucleotide, amino acid, and lipid synthesis but concurrently elevates redox pressure and methyl-donor flux. When maintained beyond adaptive windows, such coupling increases transcriptional noise and challenges chromatin stability, linking metabolic throughput directly to epigenetic remodeling [53,54,55,56].

Crucially, these architectures differ from adaptive signaling not by their molecular components, but by their temporal persistence and feedback polarity. Adaptive states preserve oscillatory regulation, enabling periodic resolution through catabolic renewal and redox rebalancing. Sustained anabolic states, by contrast, flatten oscillatory dynamics, favor feed-forward reinforcement, and reduce the capacity for recovery-mediated correction [57,58,59,60].

In the context of elite performance, these shared regulatory architectures emerge under conditions of chronic nutritional surplus, repeated high-intensity loading, or pharmacological amplification of anabolic signaling. Their relevance lies not in pathological outcome, but in illustrating how conserved control systems respond when pushed beyond oscillatory limits. This perspective situates sustained anabolic drive as a systems-level phenomenon, providing a mechanistic bridge between metabolic adaptation, redox imbalance, and downstream epigenetic drift explored in subsequent sections.

### 3.3. Epigenetic Modulation Under Metabolic Stress

The interface between metabolism and epigenetics has emerged as one of the defining discoveries of modern molecular physiology. Cellular energy flux dictates the availability of cofactors that directly modify chromatin, making metabolism not merely a supplier of energy but an active governor of gene expression. Enzymes responsible for DNA and histone modifications depend on metabolites such as S-adenosylmethionine (SAM), acetyl-CoA, α-ketoglutarate (α-KG), and nicotinamide adenine dinucleotide (NAD^+^). These metabolites serve as dynamic reporters of the nutritional and redox environment: changes in diet, exercise load, or mitochondrial efficiency immediately reverberate in the epigenome. Beyond chromatin remodeling, sustained redox imbalance may also propagate toward low-grade inflammatory tone and immune modulation through persistent ROS signaling, NF-κB activation, and altered myokine–immune cross-talk, thereby extending metabolic stress from the cellular to the systemic level [61].

In low-energy states, AMPK activation promotes NAD^+^ synthesis through the salvage enzyme NAMPT, which in turn enhances SIRT1 activity. SIRT1 deacetylates histones H3 and H4 and transcriptional regulators such as PGC-1α, FOXO, and p53, thereby promoting oxidative metabolism, autophagy, and stress resistance [62]. This AMPK–SIRT1–PGC-1α axis epitomizes a nutrient-sensing feedback that maintains chromatin compactness and genomic integrity during caloric scarcity. In contrast, nutrient oversupply generates abundant acetyl-CoA and SAM, driving histone hyperacetylation and DNA hypermethylation at promoters of oxidative and stress-response genes [63,64]. The chromatin thus “remembers” energy abundance through an anabolic epigenetic imprint.

DNA Methylation and Histone Acetylation in Exercise and Overload—exercise transiently remodels the skeletal muscle methylome. Acute endurance sessions induce hypomethylation at promoter CpG sites of metabolic regulators such as *PGC-1α*, *PDK4*, and *TFAM*, facilitating transcriptional activation and mitochondrial biogenesis. Over repeated training cycles, this hypomethylated pattern stabilizes, giving rise to the so-called *epigenetic memory* of exercise, which persists even after detraining. Such “memory” reflects a primed chromatin state that accelerates re-adaptation upon retraining—a beneficial example of epigenetic plasticity [65,66,67].

However, this flexibility has limits. When nutrient intake, supplementation, or anabolic pharmacology impose chronic mTOR activation, the methylome drifts toward hypermethylation of catabolic and antioxidant genes (*FOXO3*, *SOD2*, *CAT*) and hypomethylation of anabolic and proliferative loci (*IGF1*, *MYC*, *mTOR*) [68,69]. The resulting transcriptional asymmetry mirrors the epigenetic architecture of many cancers, in which DNA methylation suppresses checkpoints and reinforces growth signaling [70]. Histone acetylation patterns behave analogously: during caloric restriction or endurance training, reduced acetyl-CoA levels and increased SIRT activity condense chromatin and favor repair pathways, whereas chronic nutrient overload expands chromatin through HAT activation (notably p300/CBP and GCN5) and enhances transcription of biosynthetic programs [71,72].

The Sirtuin–PARP Axis: NAD^+^ Competition and Redox Imbalance—a less visible but crucial intersection between metabolism and the epigenome lies in NAD^+^ partitioning between sirtuins and poly(ADP-ribose) polymerases (PARPs). Both enzyme families consume NAD^+^, but for divergent purposes: sirtuins preserve mitochondrial function and genomic stability via deacetylation, while PARPs respond to DNA damage by poly-ADP-ribosylation, a repair signal. Under chronic oxidative stress, excessive PARP activation depletes NAD^+^, silencing sirtuin activity and collapsing the AMPK–SIRT1 axis [73]. This shift diverts energy from repair and metabolic regulation toward futile cycles of DNA damage and inflammatory signaling, a pattern documented under excessive or non-resolving exercise stress in skeletal muscle and more broadly observed in proliferative disease states [74,75]. Nutritional or pharmacological activation of AMPK (via resveratrol, berberine, or caloric restriction) can rebalance this NAD^+^ economy, restoring redox homeostasis and chromatin fidelity.

MicroRNAs and Translational Noise—microRNAs (miRNAs) represent an additional, fast-acting mechanism through which metabolic stress reshapes the adaptive landscape. Several miRNAs—notably miR-1, miR-133a, miR-206, and miR-378—coordinate muscle differentiation, mitochondrial turnover, and oxidative defense [76,77]. Endurance training typically upregulates oxidative miRNAs (miR-494, miR-181a) that enhance PGC-1α signaling, whereas resistance or overload training preferentially increases miR-378 and miR-486, which promote hypertrophy [78]. Chronic nutrient and redox stress, however, destabilize this regulatory network, producing aberrant miRNA expression such as persistent miR-21 or miR-34a overexpression, both associated with fibrotic remodeling and oncogenic pathways [79,80]. In this context, miRNAs act as both messengers and memory traces of metabolic experience—biomarkers that reflect whether adaptation remains reversible or slides toward pathology.

From Metabolic Flexibility to Epigenetic Drift—epigenetic mechanisms are inherently reversible, yet their reversibility is time- and context-dependent. Prolonged oxidative overload exhausts α-KG and NAD^+^ pools, while accumulating succinate and fumarate—competitive inhibitors of TET and Jumonji demethylases [81]. This inhibition stabilizes aberrant histone and DNA methylation, anchoring the chromatin in maladaptive configurations. Mitochondrial dysfunction amplifies this process through disrupted one-carbon flux and altered SAM/SAH ratios, leading to global methylation instability [82,83].

The term epigenetic drift describes this cumulative loss of methylation precision across the genome. In cancer and aging, drift manifests as hypervariable methylation, local hypomethylation, and transposon reactivation. In the context of high-performance sport, analogous processes might underlie the progressive reduction in adaptive potential observed in chronically overloaded athletes. Methylation noise could blunt the genomic responsiveness to training stimuli, a molecular correlate of the overtraining syndrome [84].

Such convergence of redox imbalance, NAD^+^ depletion, and methylation drift reveals a unifying framework: the same biochemical plasticity that enables human adaptation also contains the seeds of instability. The oscillation between anabolic expansion and catabolic renewal—between acetylation and deacetylation, methylation and demethylation—is the molecular rhythm of life. When that rhythm is flattened by nutritional excess or pharmacologic drive, the epigenome ceases to dance to the music of adaptation and begins to echo the static of disease.

The principal metabolic–epigenetic mechanisms are summarized in Table 2, which integrates molecular cofactors, enzymatic regulators, and evidence from elite sport to illustrate how nutrient-driven signaling shapes the reversible—or progressive—nature of epigenetic remodeling.

These findings consolidate the concept that metabolism and chromatin function as two interlocked systems, each continuously rewriting the state of the other. In elite athletes, periodic metabolic stress through training and recovery fosters a reversible form of epigenetic plasticity, allowing performance optimization without molecular instability. Yet, when this oscillation is flattened by chronic overnutrition, excessive supplementation, or pharmacological drive, the same adaptive machinery turns against itself.

Redox imbalance—↑ 8-oxo-2′-deoxyguanosine (8-oxo-dG) and/or thiobarbituric acid reactive substances (TBARS), ↓ reduced/oxidized glutathione ratio (GSH/GSSG), ↑ inflammatory redox tone.

### 3.4. Case Contexts: Elite Sport and Doping Paradigms

This section describes molecular parallels and health risks associated with performance-enhancing practices; it does not imply causation or endorsement of such interventions.

Elite sport represents an unparalleled natural laboratory for studying the extremes of human adaptation—and, occasionally, its pathologies. The metabolic ambition to optimize performance often blurs into a gray zone where physiology meets pharmacology. Recent advances in artificial intelligence and machine learning now assist in identifying performance determinants in sports, offering data-driven perspectives on training load, nutritional status and adaptive physiology [85,86]. Big-data analytical frameworks further enhance such decision-making processes by integrating multivariate performance, physiological, and contextual metrics. The interplay between training, nutrition, and doping creates an environment of sustained metabolic stimulation rarely encountered in any other context [87]. This section examines how performance-enhancing practices recapitulate the molecular features of metabolic overdrive and epigenetic destabilization described earlier.

Endurance Sports and the Hypoxic–Erythropoietic Axis—endurance sports such as professional cycling provide the most emblematic example of physiological adaptation pushed to its biochemical edge. Chronic endurance training induces a hypoxia-like signaling pattern characterized by transient stabilization of HIF-1α, activation of erythropoietin (EPO), angiogenic remodeling, and mitochondrial biogenesis [88,89]. These responses are adaptive under normal conditions, improving oxygen transport and aerobic metabolism. However, exogenous EPO administration and blood transfusions—practices widely documented in professional cycling during the 1990s and early 2000s—exaggerated this system far beyond physiological boundaries [90].

Artificially elevated hematocrit increases oxygen-carrying capacity but imposes hemodynamic strain, oxidative load, and erythroid proliferation. Sustained HIF-1α activity amplifies ROS generation and promotes a metabolic shift toward glycolysis and lactate accumulation even at rest [91]. In the long term, such pseudo-hypoxic signaling profiles are indistinguishable, at the molecular level, from tumor hypoxia responses, featuring upregulation of VEGF, GLUT1, and glycolytic enzymes [92]. Moreover, EPO itself acts as a growth and survival factor beyond erythroid cells, activating JAK2–STAT5 and PI3K–AKT pathways implicated in cancer progression [93]. While no causal link exists between EPO doping and malignancy, its signaling footprint overlaps substantially with pro-oncogenic pathways, illustrating how chronic stimulation of adaptive systems can drift toward dysregulation.

Strength Sports and the Anabolic–Epigenetic Axis—bodybuilding and other strength disciplines push the opposite side of the metabolic spectrum: sustained activation of the IGF-1–AKT–mTOR axis through resistance training, high-protein diets, and anabolic–androgenic steroids (AAS). Supraphysiological androgen exposure amplifies protein synthesis and suppresses catabolic genes via AR–mTORC1 crosstalk, while exogenous growth hormone (GH) and insulin potentiate this effect through the IGF-1 receptor [94,95,96]. In muscle tissue, these stimuli enlarge fiber cross-sectional area but also suppress autophagy, reduce mitochondrial density, and generate oxidative stress [97].

Recent molecular profiling of long-term AAS users may alter epigenetic regulation, including hypomethylation of hypertrophy-related genes and hypermethylation of stress-response pathways, consistent with the “epigenetic memory” of drug-induced adaptation [98]. Moreover, chronic AAS exposure alters liver methylation and acetylation profiles, induces gene-expression signatures that overlap with hepatocellular carcinoma profiles, without implying causal progression [99]. Case studies of elite bodybuilders who developed cardiac hypertrophy, hepatic adenomas, or renal neoplasms suggest that these effects appear molecularly traceable, though no causal inference can be drawn [100].

Nutritionally, high-protein intakes exceeding 3 g·kg^−1^·day^−1^, coupled with insulinotropic supplements and carbohydrate overfeeding, sustain mTOR activation while suppressing AMPK and SIRT1 activity [101]. The resulting anabolic lock-in mirrors the metabolic rigidity described in cancer cells: persistent mTORC1 signaling, reduced autophagic flux, and redox imbalance. This creates a cellular environment favoring DNA damage and aberrant methylation. When compounded with androgen-driven oxidative metabolism, the milieu becomes one of metabolic overdrive—high energy throughput but low regulatory elasticity.

Hormonal Manipulation and “Metabolic Doping”—beyond classical anabolic agents, a new frontier of metabolic doping targets pathways originally intended for therapeutic modulation. Thyroid hormones, β2-agonists (e.g., clenbuterol), insulin, and selective AMPK modulators (AICAR, metformin) have been misused by athletes to alter substrate utilization or enhance endurance [102,103]. These agents interfere directly with metabolic sensors and cofactor pools, producing unpredictable downstream effects on the epigenome. For instance, AICAR activates AMPK pharmacologically and can mimic the transcriptional signature of endurance training, whereas clenbuterol overstimulates adrenergic pathways and induces mitochondrial uncoupling [104,105]. Chronic manipulation of these sensors may uncouple the finely tuned oscillation between AMPK and mTOR, destabilizing redox balance and epigenetic control.

Ethical and Biomedical Implications—the molecular parallels between performance optimization and disease raise ethical and medical questions that transcend doping control. Current anti-doping frameworks focus on detection and fairness, yet the long-term health consequences of chronic metabolic stimulation remain insufficiently understood. Persistent alterations in DNA methylation, histone acetylation, and microRNA networks could represent a form of molecular scarring that outlasts pharmacological exposure. This concept reframes the notion of “clean recovery”: molecular homeostasis may take years to restore, and in some cases, residual epigenetic instability could persist indefinitely [106].

The cases of high-profile endurance and strength athletes—from those involved in EPO-era cycling to long-term steroid users in bodybuilding—exemplify how human physiology can be optimized to the edge of self-destruction. Their bodies serve as extreme testbeds where metabolic and epigenetic mechanisms of adaptation, plasticity, and degeneration converge. Understanding these processes is not merely an exercise in moral scrutiny but an opportunity to learn how the same molecular programs that sustain elite performance can, under persistent drive, erode the genomic architecture they were meant to enhance.

The integrated dynamics of oxidative metabolism and chromatin remodeling are summarized in Table 3, illustrating how redox flux and epigenetic regulation form a unified feedback architecture that defines the physiological and pathological boundaries of adaptation.

Together, these findings delineate a dynamic equilibrium in which oxidative metabolism and chromatin architecture continually inform one another. Within this circuit, redox flux is not simply a by-product of energy turnover but a transcriptional signal that encodes the metabolic state into chromatin memory. As long as this feedback remains oscillatory—alternating between activation and recovery—the system preserves its resilience. When that oscillation collapses under continuous anabolic or oxidative drive, the feedback loop loses its corrective power, locking the cell into a state of sustained activation.

At this point, adaptation becomes progressively less reversible. Regulatory coordination between energy use, redox balance, and gene regulation deteriorates, giving rise to self-reinforcing metabolic and oxidative stress. What initially supports performance optimization therefore shifts, at a mechanistic level, toward molecular features associated with reduced regulatory stability.

### 3.5. The Metabolic Overdrive Model

Across the spectrum of elite performance, metabolic adaptation is characterized by oscillations between catabolic and anabolic states—a rhythmic negotiation between energy depletion and restoration. In physiological balance, this oscillation forms a closed adaptive loop governed by reciprocal activation of AMPK and mTOR, periodic fluctuations in redox tone, and reversible epigenetic remodeling. The loop functions as an information-processing system: metabolic cues are encoded as transient chemical modifications on chromatin, which in turn regulate the next cycle of energy flux.

The Metabolic Overdrive Model is presented here as a conceptual synthesis rather than a formal mathematical or predictive model. It integrates convergent evidence from experimental studies on AMPK–mTOR antagonism, autophagy regulation, redox signaling, and NAD^+^-dependent epigenetic control, with prior theoretical work describing oscillatory and feedback-governed behavior in metabolic networks [1,3,57,58]. The purpose of this framework is not quantitative prediction, but clarification of control architecture and boundary conditions that distinguish adaptive oscillation from sustained anabolic activation.

From Oscillation to Lock-In—in the adaptive state, AMPK activation during energetic stress initiates autophagy, mitochondrial renewal, and chromatin deacetylation through SIRT1. During recovery, nutrient abundance and growth signals transiently activate mTORC1, promoting protein synthesis and tissue repair. This rhythmic succession of AMPK–mTOR–SIRT1 signaling maintains metabolic flexibility, minimizes oxidative stress, and preserves epigenetic plasticity [30,47,48].

Metabolic overdrive arises when this rhythm collapses into a static state of chronic anabolism. Persistent nutrient or hormonal stimulation suppresses AMPK checkpoints, silences sirtuins, and locks mTORC1 in constitutive activation [30,48]. The system transitions from oscillation to saturation—energy continues to flow, but regulatory feedback weakens. Elevated acetyl-CoA, NADH/NAD^+^ imbalance, and ROS accumulation feed forward into histone hyperacetylation and aberrant DNA methylation [107]. The epigenome, once adaptive, becomes entrained to a pathological steady state: hypertranscriptional but inflexible.

Formal view—The dynamics can be approximated as a loss-of-oscillation (Hopf-like) bifurcation: as energetic input (nutrient/hormonal drive) increases, the amplitude of the AMPK–mTOR oscillation diminishes toward zero; beyond a critical input threshold, the oscillatory attractor disappears and the system converges to a single high-anabolic steady state (mTORC1-dominant “lock-in”). This statement is a conceptual analogy, consistent with emergent oscillatory/steady-state behaviors reported in dynamic models of the AMPK–ULK1–mTOR regulatory triangle and the broader mTOR signaling network [57,58,59,60].

**Figure 3:**
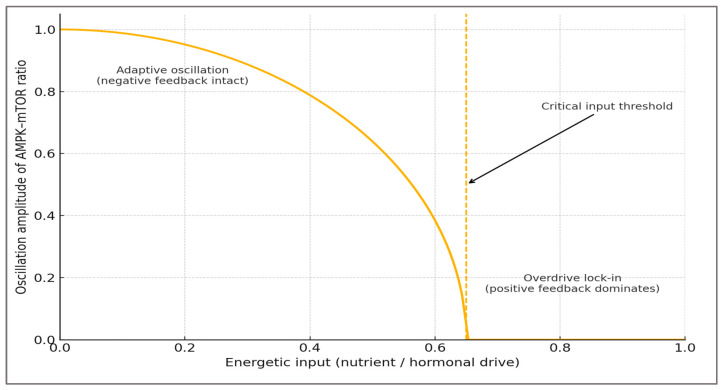
Bifurcation schematic of the Metabolic Overdrive Model. Increasing energetic or hormonal input reduces the oscillation amplitude of the AMPK–mTOR ratio. Beyond the critical input threshold (dashed line), the system collapses to a high-anabolic steady state (“lock-in”) [59,60].

This conceptual transition is illustrated in Figure 3, which summarizes the loss-of-oscillation threshold separating adaptive AMPK–mTOR cycling from anabolic lock-in.

Bridge to architecture—Operationally, the threshold in figure maps onto the Box 1 criteria: persistence >24 h of ↑p-S6K1/4E-BP1 with ↓AMPK Thr172-P, accompanied by NAD^+^ depletion/↑PARylation and redox/epigenetic drift. This is the measurement logic summarized in the last table.

**Figure 4:**
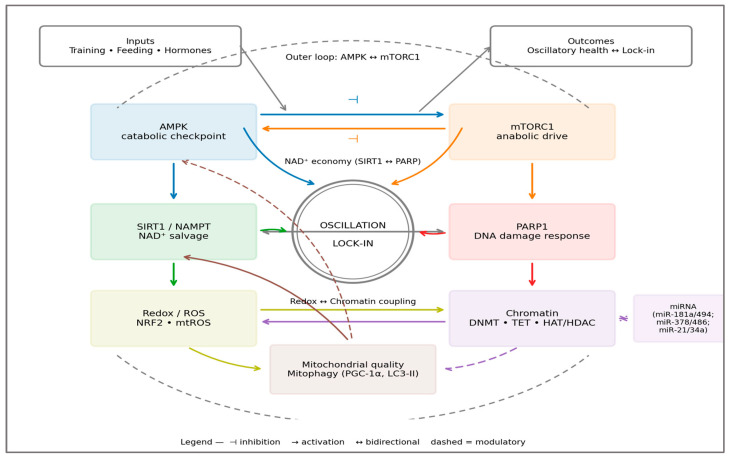
Dual-loop architecture of the Metabolic Overdrive Model. The diagram illustrates the systems-level organization of exercise-induced adaptation as a dual-loop control architecture. The outer loop represents the oscillatory antagonism between AMPK and mTORC1 across cycles of training, feeding, and recovery. Under adaptive conditions, energetic stress during exercise activates AMPK, promoting catabolic checkpoints, autophagy, and mitochondrial renewal, while subsequent nutrient availability transiently reactivates mTORC1 to support protein synthesis and structural remodeling. The adaptive signature is the antiphase relationship between AMPK and mTORC1 activity (peak and nadir separation). The inner loop depicts redox–epigenetic coupling, centered on the NAD^+^ economy governed by the balance between SIRT1-mediated deacetylation and PARP-dependent NAD^+^ consumption. Transient ROS pulses and controlled redox signaling act as informational inputs that modulate chromatin state through reversible DNA methylation, histone acetylation, and non-coding RNA regulation. Metabolic Overdrive emerges when persistent energetic, nutritional, or hormonal drive compresses the oscillatory window, leading to sustained mTORC1 activation, suppressed AMPK signaling, disruption of the NAD^+^ balance, and epigenetic drift. In this lock-in state, feedback polarity shifts from negative to positive, reducing recovery capacity and system resilience. Solid arrows denote dominant regulatory directionality, dashed arrows indicate modulatory interactions, and inhibitory connections are shown as blunt-ended lines. The model emphasizes that Metabolic Overdrive represents a continuum, with functional outcomes determined by the system’s ability to re-establish rhythmic alternation. Sources: AMPK–mTOR antagonism and autophagy coupling [1,19,28,29]; NAD^+^/SIRT1/PARP coupling [62,73]. Color coding is used to visually distinguish functional modules within the dual-loop architecture and to clarify directional interactions between metabolic and redox-epigenetic components.

The Closed-Loop Architecture of Adaptation—Figure 4 (conceptual diagram) illustrates the dual-loop control underpinning these dynamics: an outer AMPK–mTOR oscillator across training/feeding and an inner redox–epigenetic loop via the SIRT1–PARP NAD^+^ economy. When negative feedback is intact, the loops remain antiphased and resilient; under chronic drive, feedback polarity shifts toward positive, predisposing to lock-in.

Together, the outer AMPK–mTOR and inner redox–epigenetic circuits form a closed-loop system in which metabolic sensing and chromatin remodeling are dynamically coupled.

Redox-Epigenetic Coupling and Systemic Instability—at the biochemical level, overdrive couples redox imbalance to epigenetic drift. Excess ROS oxidize 5-methylcytosine and histone residues, impairing TET- and Jumonji-mediated demethylation [103]. NAD^+^ depletion through PARP overactivation reduces SIRT-dependent chromatin silencing, compounding transcriptional noise. As feedback fidelity deteriorates, the epigenome begins to store the metabolic history of stress—a persistent “memory” of overload. Biomechanical asymmetry during repetitive effort may further amplify local metabolic load and redox stress, providing a physical correlate of systemic overdrive [108]. Such molecular scars explain the lingering performance decrements and altered recovery profiles seen in chronically overtrained athletes [109].

From Adaptation to Pathology—in its terminal form, metabolic overdrive recapitulates the molecular hallmarks of tumorigenesis: constitutive mTOR signaling, defective autophagy, mitochondrial hyperpolarization, and aberrant methylation [110,111]. Yet the model is not a statement of equivalence between athletic performance and cancer, but of shared architecture—both are systems pushed beyond adaptive elasticity. The lesson is systemic: human performance is limited not by energy supply, but by the capacity to restore homeostatic oscillation after each perturbation. Within sport science, a closely related but non-oncologic phenotype of non-resolving stress is overreaching/overtraining, where performance decrements persist alongside neuroendocrine and autonomic disruption; classic and consensus frameworks emphasize that the key failure mode is inadequate recovery and an inability to re-establish homeostasis [112,113,114].

Implications—the *Metabolic Overdrive Model* redefines performance physiology as a dynamic equilibrium rather than a linear progression. It integrates nutrient sensing, redox homeostasis, and epigenetic regulation into one mechanistic continuum, uniting the biology of adaptation, aging, and disease. By identifying the molecular inflection point between beneficial stress and chronic overload, this framework offers a language to discuss both the promise and the peril of metabolic enhancement. In elite sport—and perhaps in life itself—resilience may depend less on how much energy we can generate than on how gracefully we can oscillate between expenditure and renewal. In practice, this favors periodized nutrition and judicious supplement use (including widely used agents such as caffeine and polyphenols like resveratrol) that supports redox recovery and the re-engagement of AMPK, rather than a strategy of constant post-exercise anabolic reinforcement [115,116,117,118].

To operationalize the Metabolic Overdrive Model, the key regulatory axes—nutritional, redox, and epigenetic—can be compared between adaptive and pathological configurations. This framework summarizes how temporal coordination across these domains sustains performance resilience, whereas persistent activation transforms feedback into self-reinforcing instability.

Table 4 presents a comparative synthesis of molecular, functional, and systemic features characterizing the transition from physiological adaptation to metabolic lock-in, integrating evidence from elite sport contexts.

Together, these patterns reveal that high performance and molecular fragility are separated not by intensity but by rhythm. When oscillatory checkpoints fail, systems designed for renewal become trapped in activation. The restoration of resilience, therefore, lies not in suppressing energy flux, but in reestablishing periodicity—the cellular capacity to alternate between catabolic repair and anabolic growth without losing feedback integrity.

### 3.6. Testable Predictions and Biomarkers

#### 3.6.1. Rationale for Biomarker Selection

The biomarker panel proposed in this framework was selected to capture distinct regulatory layers of the Metabolic Overdrive Model, spanning energy sensing (AMPK–mTOR balance), redox and NAD^+^ economy, and downstream epigenetic remodeling. Priority was given to readouts that (1) directly reflect pathway activity rather than downstream outcomes, (2) display time-dependent behavior across recovery windows, and (3) have been previously validated in exercise or metabolic stress contexts. Where tissue specificity is required, skeletal muscle markers (e.g., AMPK Thr172 phosphorylation, autophagy-related proteins) are cited as reference standards based on existing exercise-biopsy literature; however, the present framework does not propose muscle biopsy as a routine or required method and acknowledges that biopsies are appropriate only in ethically approved, informed-consent research settings. Instead, peripheral blood mononuclear cells and plasma-based measures are emphasized as translational surrogates for NAD^+^ metabolism, redox status, and circulating microRNAs. Table 5 summarizes the selected biomarkers, their specimen type, expected direction of change under sustained anabolic dominance, and the strength of supporting evidence [6,17,30,62,73,80]. Human biopsy data showing induction of autophagy-related genes after ultraendurance exercise support the use of autophagy flux markers as mechanistic anchors [119]. Likewise, PBMC-based DNA repair and PARP activity assays after acute exercise provide a rationale for including PARylation and NAD^+^ readouts in minimally invasive designs, consistent with PARP–sirtuin competition for NAD^+^ [120,121]. The broader exercise-metabolism framework and signaling kinetics that informed our timing assumptions are discussed in authoritative syntheses of skeletal muscle adaptation [122,123].

#### 3.6.2. Testable Predictions (P1–P3)

Based on the AMPK–mTOR antagonism, the SIRT1–PARP NAD^+^ economy, and redox–epigenetic coupling summarized in Section 3.1, Section 3.2 and Section 3.3 and operationalized in Box 1 and Table 5, we propose the following falsifiable predictions:

**P1.**  Persistent anabolic drive flattens the AMPK–mTOR rhythm. After heavy training plus high nutrient/hormonal load, p-S6K1/4E-BP1 remain elevated >24 h while AMPK Thr172-P is depressed, indicating a shift toward sustained mTORC1 dominance and reduced autophagy. Readouts: AMPK Thr172-P, p-S6K1, p-4E-BP1, LC3-II, p62. Rationale: Section 3.1 and Box 1.

**P2.**  Redox stress drives an NAD^+^ sink and epigenetic drift. Overreaching under anabolic excess increases PARP activity (PARylation), lowers NAD^+^ and SIRT1 activity, and biases chromatin toward ↑H3K9ac with ↓TET activity/5hmC. Readouts: NAD^+^/NADH, PARylation, SIRT1 activity, H3K9ac, 5hmC. Rationale: Section 3.3 and Section 3.6.

**P3.**  Periodic AMPK restoration re-establishes oscillation. Interventions that intermittently activate AMPK (e.g., fasted endurance, time-restricted feeding, polyphenol-driven AMPK activation) normalize NAD^+^, reduce PARylation/ROS, and reverse acetylation/methylation drift. Readouts: AMPK Thr172-P, NAD^+^, PARylation, H3K9ac, LC3-II. Rationale: Section 3.1, Section 3.2 and Section 3.3 and Table 5.

Measurement window: 24–48 h post-stimulus phase used to distinguish adaptive oscillation from lock-in dynamics. These hypotheses are non-causal claims but operational tests of the Metabolic Overdrive Model.

To operationalize predictions P1–P3, we map human-assayable readouts onto the 0–48 h recovery window, separating fast redox/NAD^+^ shifts from slower structural signals. This clarifies which endpoints are expected to move immediately (T_0_–T_1_) versus those that resolve or diverge at T_2_–T_3_, where oscillation versus lock-in becomes distinguishable.

**Figure 5:**
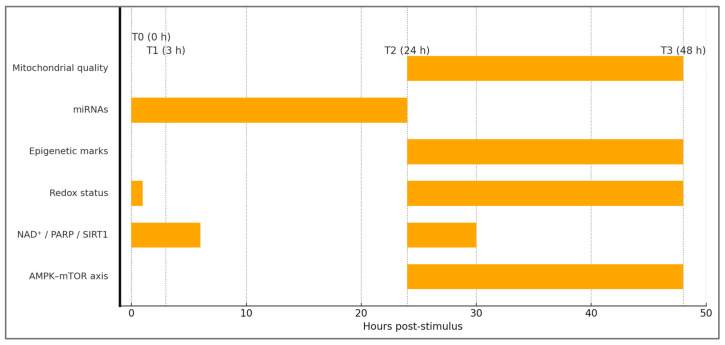
Operational biomarker timeline (0–48 h) and preferred sampling windows. Bars denote recommended collection windows: AMPK–mTOR axis (24–48 h), NAD^+^/PARP/SIRT1 (0–6 h and ~24–30 h), Redox status (0–1 h and 24–48 h), Epigenetic marks (24–48 h), miRNAs (0–24 h), Mitochondrial quality (24–48 h). Dashed vertical markers indicate T_0_ (0 h), T_1_ (3 h), T_2_ (24 h), T_3_ (48 h). Windows are conceptual and harmonized with Table 5 and the protocol in Box 2. Timing rationale is derived from published post-exercise signaling kinetics and biomarker studies [6,30,73,74,75,76,77,78,80].

Preferred sampling windows across T_0_–T_3_ are visualized in Figure 5, aligning predictions P1–P3 with assay timing.

Operational biomarkers, preferred sampling windows and the strength of evidence supporting each axis are summarized in Table 5.

Interpretation of Table 5—The proposed biomarker panel is intentionally tiered. Tissue-based readouts (e.g., muscle AMPK Thr172-P, p-S6K1/p-4E-BP1, LC3-II/p62 under lysosomal inhibition) serve as biological anchors for mechanistic validation in controlled research settings. For routine or field-feasible testing, Table 5 emphasizes minimally invasive surrogates (PBMC and plasma measures of NAD^+^/PARylation, redox status, and circulating microRNAs) that can be repeatedly sampled across training cycles. This distinction clarifies that “biomarker panel” here refers primarily to circulating and PBMC-based measures, with muscle markers reserved for hypothesis verification [6,17,73,74,80].

#### 3.6.3. Pre-Analytical Considerations and Feasibility

Pre-analytical standardization is critical because AMPK–mTOR phosphorylation, NAD^+^/NADH ratios, redox markers, and circulating miRNAs display strong time-of-day, feeding, and training-load dependence. At minimum, studies should standardize: (1) the exercise stimulus (work performed, intensity, and muscle groups), (2) post-exercise nutrition (timing and macronutrient composition), and (3) sampling time relative to exercise and feeding (T_0_–T_3_ in Figure 5). A within-subject repeated-measures design is preferred to reduce inter-individual baseline variability [6,30,73,74].

For blood-based assays, practical steps include rapid processing (ideally ≤30–60 min), consistent anticoagulant choice, and storage conditions (e.g., plasma/serum aliquots at −80 °C) to minimize degradation and hemolysis artifacts. PBMC isolation introduces additional variability; therefore, reporting of isolation method, cell counts/viability, and normalization strategy (per cell number or protein content) is recommended. For miRNA-based readouts, hemolysis controls and the use of stable reference miRNAs improve interpretability. These considerations should be reported explicitly as they determine whether apparent “lock-in” patterns reflect biology or sample handling [73,74,75,76,77,78,80].

Ethical feasibility also constrains tissue-based measurements. Muscle biopsy remains the reference method for interrogating local phosphorylation, autophagy flux, and chromatin marks in skeletal muscle; however, it should be limited to well-justified laboratory studies with ethics approval and informed consent. The present framework therefore prioritizes a blood-first approach (PBMC/plasma), with optional biopsy used only for mechanistic validation where appropriate [17,30,73].

Box 2 **Practical protocol for testing the Metabolic Overdrive hypothesis in humans.**

Study design (recommended): within-subject, repeated-measures time series across a defined training microcycle, using a standardized “metabolic challenge” (e.g., a heavy session followed by a controlled feeding strategy) and sampling at T_0_ (pre), T_1_ (~3 h), T_2_ (~24 h), T_3_ (~48 h).

Core specimens (field-feasible): venous blood for plasma/serum and PBMC at all time points; optional urine (oxidative markers) and saliva (cortisol) as supportive context. Muscle biopsy is optional and should be restricted to mechanistic validation studies with ethics approval.

Primary readouts mapped to predictions: P1 (AMPK–mTOR rhythm): p-S6K1/p-4E-BP1 and AMPK Thr172-P (muscle if available; PBMC as exploratory), plus autophagy markers (LC3-II, p62) where flux can be assessed. P2 (NAD^+^ sink and epigenetic drift): PBMC/plasma NAD^+^/NADH, PARylation and/or PARP activity, SIRT1 activity proxies, and selected chromatin marks (e.g., H3K9ac, 5hmC) in PBMC. P3 (reversibility): changes in these axes after an AMPK-restoring intervention (e.g., fasted low-intensity session or TRF block) within the same athlete.

Decision logic (operational): classify a candidate “overdrive” episode when ≥3 Box 1 criteria persist at ≥24 h (T_2_) and fail to normalize by 48–72 h, with particular emphasis on sustained p-S6K1/p-4E-BP1 together with suppressed AMPK Thr172-P and a concordant NAD^+^/PARylation shift. Report concurrent training load, dietary intake, sleep, illness/inflammation, and supplementation to contextualize biomarker interpretation.

Quality control: pre-register the sampling schedule, standardize processing and storage, include hemolysis controls for miRNAs, and analyze trajectories as time series (not single time points). Where feasible, compute a composite “Adaptive Oscillation Index” from normalized AMPK–mTOR and NAD^+^/PARylation features to summarize oscillation vs lock-in behavior across T_0_–T_3_.

## 4. Synthesis and Outlook

This review positions exercise adaptation as a time-domain phenomenon: benefits emerge when energetic stress and nutrient recovery remain temporally separable, allowing AMPK- and mTORC1-dependent programs to alternate rather than overlap. By integrating AMPK–mTOR antagonism with NAD^+^/redox/epigenetic coupling, the Metabolic Overdrive Model frames the adaptive ceiling as a loss-of-oscillation problem: when anabolic signaling fails to resolve within the recovery window, feedback fidelity declines and the system drifts toward an mTORC1-dominant steady state (“lock-in”). The practical value of this synthesis is not a clinical claim, but an explicit set of operational readouts and sampling windows (Box 1 and Box 2, Figure 5, Table 5) that enable empirical falsification in humans. This framing is consistent with recent syntheses on exercise-linked mitochondrial/epigenetic remodeling and body-composition-related epigenetic responses [124,125], and with integrative views of the molecular regulation of skeletal muscle adaptation [122].

Practical implications—For athlete monitoring and nutrition/training periodization, the model suggests prioritizing restoration of oscillation (resolution of mTORC1 signals and reactivation of AMPK checkpoints) over maximizing continuous anabolic tone. Because repeated tissue sampling is often infeasible, the proposed testing strategy is “blood-first”: PBMC/plasma markers of NAD^+^ economy, PARylation, redox status, and selected miRNAs are used for repeated surveillance, while muscle biopsy is reserved for mechanistic validation in ethically approved laboratory studies. The cancer literature is used as a mechanistic comparator of sustained anabolic signaling and epigenetic drift, not as an implied outcome of elite sport. At the mechanistic level, endurance-driven improvements in the oxidative phenotype and PGC-1α-mediated anti-inflammatory signaling illustrate how restoring catabolic renewal can stabilize redox tone while preserving performance adaptations [123,126].

Limitations and Future Directions—While this review offers a mechanistic synthesis grounded in established molecular principles, it remains a conceptual construct requiring empirical validation. Most data supporting the oscillatory model derive from indirect or cross-sectional evidence. Future studies should employ longitudinal multi-omics approaches—integrating transcriptomic, metabolomic, and methylomic time-series—to track molecular cycles of AMPK–mTOR activation, NAD^+^ flux, and redox oscillation during structured training interventions. Computational modeling could further identify energetic thresholds or “bifurcation points” marking the transition from adaptive to maladaptive states. Constraint-based and kinetic modeling approaches, together with metabolomics-integration frameworks, provide a practical starting point for formalizing the proposed thresholds and interpreting multi-omics time series [127,128]. In parallel, incorporating multi-trait genetic panels may help contextualize inter-individual variability in adaptive ceilings and recovery capacity [129].

Elite performance provides an ideal setting for this inquiry. The same interventions used to maximize adaptation—nutritional periodization, recovery modulation, and exercise dosing—can serve as experimental tools for testing system stability. Integrating molecular assays with performance metrics may reveal biomarkers of oscillatory health, guiding safer performance enhancement and long-term metabolic preservation.

Importantly, features consistent with the Metabolic Overdrive phenotype may be transiently expressed during periods of intensified training or competitive peak, particularly in elite athletes. In such contexts, short-lived elevations in anabolic signaling, redox flux, and biosynthetic activity are likely to support performance optimization and adaptive remodeling. The model does not frame these features as inherently maladaptive; rather, it distinguishes between adaptive, time-limited overdrive and chronic, non-resolving overdrive, in which sustained anabolic dominance, redox imbalance, and impaired recovery undermine long-term adaptation. Accordingly, Metabolic Overdrive is conceptualized as a continuum, with performance outcomes determined by the system’s capacity to re-establish oscillatory control following periods of intensified metabolic drive.

## 5. Conclusions

Human performance emerges from a dynamic equilibrium between energy generation and molecular repair. The *Metabolic Overdrive Model* proposed here reframes this equilibrium as an oscillatory dialogue between nutrient sensing, redox regulation, and epigenetic plasticity. Within this loop, AMPK and mTOR act not as antagonists but as temporal partners, orchestrating periodic cycles of depletion and renewal that sustain adaptation. When this rhythm collapses—through nutritional excess, pharmacologic stimulation, or chronic overload—the system enters metabolic overdrive: an energetically amplified but informationally unstable state.

This framework unites the physiology of performance with the biology of instability. The same molecular circuits that enable hypertrophy, angiogenesis, and resilience under stress can, when persistently activated, erode redox homeostasis, silence autophagy, and imprint maladaptive epigenetic marks. The border between adaptation and pathology is therefore not structural but rhythmic—defined by whether the organism can restore oscillation after each pulse of stress.

Understanding performance through this lens moves the field beyond simple narratives of training load or nutrient intake toward a systems view of resilience. It invites a future in which athlete health is monitored not only through hormonal or metabolic markers but also through signatures of epigenetic flexibility and redox rhythm. Protecting the capacity to oscillate—to alternate between anabolism and repair—may prove to be the true foundation of both sustained exercise adaptation and performance stability.

## Figures and Tables

**Table 1:** Nutritional signaling pathways and their integrated roles in metabolic adaptation.

Pathway/Component	Nutritional or Energetic Trigger	Upstream Regulator(s)	Primary Molecular Effect	Downstream Target(s)	Adaptive Role (Physiological Context)	Potential Dysregulation (Overdrive State)
**AMPK**	Energy deficit, exercise, fasting	AMP/ATP ratio, LKB1	Activates catabolic fluxes; inhibits mTORC1	ACC, ULK1, PGC-1α	Enhances mitochondrial biogenesis, endurance, autophagy	Chronic suppression → anabolic dominance, insulin resistance
**mTORC1**	Amino acids (leucine), insulin, IGF-1	PI3K–AKT, Rheb, Rag GTPases	Stimulates protein synthesis via p70S6K and 4E-BP1	Ribosomal proteins, eIF4E	Promotes muscle growth and recovery	Persistent activation → oxidative stress, epigenetic drift
**SIRT1**	NAD^+^ levels, caloric restriction	AMPK, NAMPT	Deacetylates transcriptional regulators (PGC-1α, FOXO)	PGC-1α, p53, NF-κB	Increases oxidative metabolism and stress resistance	NAD^+^ depletion → loss of redox control and mitochondrial decline
**IGF-1/AKT axis**	Protein intake, GH, insulin	GH/IGF-1 signaling	Activates mTORC1, inhibits AMPK	FOXO, TSC2, GSK3β	Supports hypertrophy and tissue regeneration	Chronic activation → reduced autophagy, oncogenic signaling
**Leucine–AMPK–mTOR crosstalk**	Branched-chain amino acids	Regulator complex	Coordinates anabolism–catabolism balance	mTORC1, AMPK	Fine-tunes training–fuel coupling, metabolic flexibility	Excess BCAA intake → insulin desensitization, ROS accumulation
**NAD^+^/PARP balance**	Oxidative load, DNA repair demand	PARP1, SIRT1	Competes for NAD^+^ substrate	SIRT1, PGC-1α	Links redox balance to mitochondrial maintenance	PARP hyperactivation → NAD^+^ depletion, energy collapse
**FOXO transcription factors**	Energy deficit, oxidative stress	AMPK, SIRT1, AKT	Regulate autophagy, antioxidant enzymes	Catalase, MnSOD, LC3	Antioxidant defense and longevity	AKT-mediated inhibition → reduced oxidative protection

Notes: Nutritional signaling integrates energetic and anabolic cues through AMPK–mTOR–SIRT1 coordination, maintaining metabolic flexibility and cellular homeostasis [1,3,6,10,17,19,30,31,33,34,38,39,40].

**Table 2:** Epigenetic regulation under metabolic stress: mechanisms linking redox imbalance, nutrient flux, and adaptive instability.

Epigenetic Layer	Primary Metabolic Cofactor or Pathway	Key Enzymes/ Regulators	Physiological Role (Adaptive)	Pathological Outcome (Overdrive State)	Representative Molecular Targets	Evidence from Elite Sport	Potential Interventions/ Modulators
**DNA methylation**	SAM/ one-carbon metabolism	DNMT1, TET1–3, MTHFR, MTR		Hypermethylation of antioxidant genes; hypomethylation of anabolic/proliferative loci; methylation drift	*PGC-1α*, *PDK4*, *TFAM*, *FOXO3*, *MYC*	Endurance athletes show promoter hypomethylation of *PGC-1α* and *TFAM* after repeated training cycles	Folate and methionine balance, caloric restriction, AMPK activation
**Histone acetylation**	Acetyl-CoA, NAD^+^/AMPK–SIRT1 axis	SIRT1, p300/CBP, GCN5, HDACs	Chromatin compaction under energy deficit; regulation of repair and mitochondrial genes	Hyperacetylation and persistent transcription of anabolic programs	*H3K9ac*, *H4K16ac*, *PGC-1α*, *p53*	Training fasted or under caloric restriction enhances SIRT1 activity and deacetylation	Resveratrol, curcumin, exercise–fasting cycles
**Histone and DNA demethylation**	α-KG/TCA flux	TETs, Jumonji demethylases	Removal of repressive marks; mitochondrial-nuclear communication	α-KG depletion, succinate/fumarate inhibition of demethylases	*H3K27me3*, *H3K9me3*, *IDH2*, *SDH*	Overreaching phases show transient TET up-regulation linked to oxidative flux	Endurance exercise, antioxidant restoration
**NAD^+^-dependent regulation**	NAD^+^ salvage, PARP vs. sirtuins	PARP1, SIRT1, NAMPT	Balanced DNA repair and mitochondrial biogenesis	PARP overactivation → NAD^+^ depletion, SIRT1 silencing, redox collapse	*PGC-1α*, *FOXO*, *PARP1*, *SIRT1*	Overtraining decreases muscle NAD^+^ and SIRT1, paralleling redox fatigue	Caloric restriction, AMPK activators, NAD^+^ precursors
**MicroRNA regulation**	Energy/redox-dependent transcription	miR-1, miR-21, miR-34a, miR-486, miR-494	Fine-tuning of hypertrophy, oxidative capacity, stress defense	Aberrant miRNA expression driving fibrosis and oncogenic signaling	*miR-21*, *miR-34a*, *miR-133a*, *miR-206*	Endurance ↑ miR-181a, miR-494; resistance ↑ miR-378, miR-486; overtraining ↑ miR-21	Training modulation, antioxidant support
**One-carbon and methyl donor flux**	Methionine–folate cycle	MAT2A, BHMT, SHMT1/2	Maintenance of methyl balance for DNA/histone regulation	SAM/SAH imbalance, global hypermethylation, redox-linked drift	*DNMT3A*, *HMTs*, *MTHFD1L*	High-protein diets alter plasma methionine and SAM/SAH ratios in athletes	Controlled protein intake, B-vitamin support

Notes: Metabolic stress remodels chromatin through energy-derived cofactors that couple redox status to gene regulation. In elite sport, these same epigenetic pathways mediate both resilience and exhaustion, defining the molecular border between adaptation and drift [7,8,9,10,11,12,63,64,65,66,67,68,69,70,71,72,73,74,75,76,77,78,79,80,81,82].

**Table 3:** The redox–epigenetic feedback loop: a systems model linking oxidative metabolism, chromatin regulation, and adaptive limits.

System Component	Primary Molecular Driver	Key Sensors or Enzymes	Physiological Feedback (Adaptive)	Pathological Feedback (Overdrive State)	Representative Molecular Signature	Potential Modulators/Countermeasures
**Mitochondrial ROS generation**	Electron transport flux, NADH/NAD^+^ ratio	Complex I–III, NOX, SOD2	ROS act as signaling molecules activating AMPK and antioxidant genes	Chronic ROS accumulation leads to mtDNA damage, lipid peroxidation, and nuclear stress signaling	↑ ROS, ↑ SOD2, ↑ NRF2	Endurance training, antioxidant periodization, redox-adaptive nutrition
**NAD^+^ metabolism**	NAMPT salvage pathway, PARP activity	NAMPT, PARP1, SIRT1	Balanced NAD^+^ use supports DNA repair and mitochondrial biogenesis	PARP overactivation depletes NAD^+^, silencing SIRT1 and impairing repair	↓ NAD^+^, ↓ SIRT1, ↑ PARylation	Caloric restriction, resveratrol, niacinamide, AMPK activation
**AMPK–SIRT1–PGC-1α axis**	Energy sensing, NAD^+^/AMP ratio	AMPK, SIRT1, PGC-1α	Enhances oxidative metabolism, mitophagy, and chromatin integrity	Collapse of AMPK–SIRT1 feedback causes autophagy failure and metabolic rigidity	↑ PGC-1α, ↑ LC3-II, ↓ FOXO	Exercise-induced AMPK activation, fasting cycles
**DNA and histone modifications**	SAM/SAH ratio, α-KG availability	DNMTs, TETs, HDACs, HATs	Dynamic methylation/acetylation maintains gene expression flexibility	Methylation drift and histone hyperacetylation stabilize maladaptive transcription	↑ H3K9ac, ↓ TET activity, ↑ DNMT1	Balanced methyl donor intake, one-carbon flux restoration
**Inflammatory redox signaling**	NF-κB, NLRP3, cytokine ROS loops	NF-κB, IL-6, TNF-α	Transient activation supports repair and immune remodeling	Chronic activation sustains oxidative stress and metabolic block	↑ NF-κB, ↑ IL-6, ↑ TNF-α	Polyphenols, omega-3s, anti-inflammatory recovery
**Epigenetic memory and drift**	ROS/NAD^+^-dependent enzyme regulation	SIRT1, PARP1, DNMT1, miRNAs	Transient chromatin remodeling encodes adaptive responses	Persistent oxidative stress leads to irreversible epigenetic drift	Hypomethylated oncogenes, hyperacetylated histones	Controlled recovery, antioxidant therapy, NAD^+^ support
**System-level outcome**	Redox–epigenetic coupling	Integrated signaling through AMPK–mTOR–SIRT1	Self-limiting oscillation ensures resilience	Feedback saturation locks system in pathological anabolism	↓ AMPK, ↑ mTOR, ↑ ROS	Nutritional periodization, training load modulation

Notes: Redox flux and chromatin dynamics form a self-reinforcing circuit that determines the trajectory of cellular adaptation. When balanced, this loop promotes renewal; when sustained beyond control, it accelerates molecular entropy and epigenetic drift [12,32,38,44,52,61,62,73,80,82].

**Table 4:** The Metabolic Overdrive Framework: comparative features of adaptive and pathological states across metabolic axes.

Regulatory Axis	Adaptive (Oscillatory) State	Overdrive (Lock-In) State	Representative Molecular Indicators	Functional Outcome (Physiological/Clinical)	Evidence or Relevance in Elite Sport	Reversibility Potential
**Nutritional signaling (AMPK–mTOR)**	Alternating activation maintains energy balance and anabolic–catabolic cycling; mTOR activity transient and self-limiting.	Chronic nutrient or hormonal stimulation suppresses AMPK; mTORC1 locked in constitutive activation.	Adaptive: ↑ AMPK (Thr172-P), oscillatory ↑ mTOR (Ser2448-P); Overdrive: ↑ S6K1, ↓ AMPK.	Efficient recovery, balanced hypertrophy, metabolic flexibility.	Observed in endurance vs. bulking athletes; chronic protein or insulin use suppresses AMPK signaling.	High—restored through fasting, caloric periodization, AMPK activators.
**Redox homeostasis (ROS–NAD^+^ balance)**	Controlled ROS pulses activate NRF2–SIRT1 defense; NAD^+^ recycling sustains redox tone.	ROS accumulation exceeds detox capacity; PARP activation depletes NAD^+^, silencing SIRT1.	Adaptive: ↑ NRF2, ↑ SIRT1, stable NAD^+^/NADH; Overdrive: ↑ PARP1, ↓ NAD^+^, ↑ oxidative adducts.	Enhanced mitochondrial efficiency and repair capacity.	Seen in overtraining and altitude exposure; redox imbalance linked to delayed recovery and fatigue.	Moderate—restored with NAD^+^ boosters, antioxidant periodization.
**Epigenetic regulation (chromatin remodeling)**	Reversible histone deacetylation and DNA demethylation maintain transcriptional flexibility; stress memory remains functional.	Hyperacetylation and aberrant methylation fix anabolic programs; transcriptional noise accumulates.	Adaptive: ↑ TET1–3, ↑ HDACs; Overdrive: ↑ DNMT1, ↑ HATs, ↑ H3K9ac.	Improved adaptation memory, genomic stability.	Exercise-induced hypomethylation of *PGC-1α* and *TFAM* vs. hypermethylation under chronic overload.	Variable—reversible early, lost under prolonged overload.
**Mitochondrial dynamics**	Balanced fusion–fission and autophagic recycling sustain bioenergetic quality.	Hyperpolarization, impaired mitophagy, ROS leakage disrupt energy homeostasis.	Adaptive: ↑ PGC-1α, ↑ MFN2, ↑ LC3-II; Overdrive: ↑ DRP1, ↑ ROS.	Optimal endurance and energy turnover.	Endurance training enhances mitophagy; chronic supplementation or doping impairs mitochondrial turnover.	Moderate—restored through recovery and redox normalization.
**System-level behavior**	Oscillatory negative feedback ensures renewal after stress; system remains dynamically stable.	Positive feedback loops reinforce anabolic and oxidative stress; system enters chaotic saturation.	Adaptive: oscillatory AMPK/mTOR ratio; Overdrive: flattened rhythm, ↑ entropy.	Resilient adaptation, sustained performance.	Elite athletes under chronic load exhibit reduced HRV and hormonal adaptability—biomarkers of systemic lock-in.	Low—requires full metabolic reset through deloading and restoration cycles.

Notes: The final form of the Metabolic Overdrive Framework integrates molecular dynamics with evidence from elite performance physiology. The collapse of oscillatory regulation—observable in both molecular biomarkers and functional markers such as fatigue, hormonal rigidity, and impaired recovery—defines the biochemical signature of adaptive exhaustion [1,3,6,10,17,19,30,31,32,33,34,38,39,40,61,62,63,64,65,66,67,68,69,70,71,72,73,80,87,88,89,90,91,95,96,98,99,100,110,111].

**Table 5:** Level of evidence and operational biomarkers for the Metabolic Overdrive Model.

Mechanistic Axis	Primary Human Readouts (Assay & Specimen)	Predicted Direction in Overdrive	Evidence Base (Cell/Animal/Human)	Reversibility Signal (Periodic AMPK)	Preferred Sampling Window
**AMPK–mTOR balance**	AMPK Thr172-P (WB/ELISA; muscle or PBMCs), p-S6K1 (Thr389)/p-4E-BP1 (Ser65) (WB), LC3-II/p62 (autophagic flux with lysosomal inhibition)	↓ AMPK Thr172-P; ↑ p-S6K1/4E-BP1; ↓ autophagic flux	Strong (cells/rodents) + human acute & training blocks	↑ AMPK Thr172-P; normalization of p-S6K1; ↑ LC3-II	24–48 h post-stimulus, spanning feeding
**NAD^+^ economy (SIRT1–PARP)**	NAD^+^/NADH (enzymatic cycling; muscle/PBMCs), PARylation (WB), SIRT1 activity (fluor./ELISA)	↓ NAD^+^; ↑ PARylation; ↓ SIRT1 activity	Moderate–strong (cells/rodents) + human acute (exercise/irradiation)	↑ NAD^+^; ↓ PARylation; ↑ SIRT1	0–6 h and 24 h post-stimulus
**Redox** **status**	8-oxo-dG (DNA damage), GSH/GSSG, TBARS/MDA, SOD2/Catalase (WB/activity)	↑ oxidative adducts; ↑ inflammatory redox tone	Strong across models; robust human acute	↓ adducts; normalization of GSH/GSSG	Immediately post + 24–48 h
**Epigenetic layer**	H3K9ac (ChIP-WB), 5mC/5hmC (LC-MS/MS; targeted bisulfite), TET activity, DNMT1; target loci: PGC-1α, TFAM, PDK4	↑ H3K9ac; ↓ TET/5hmC at oxidative loci; ↑ DNMT1; methylation drift	Moderate (cells/rodents) + human acute/longitudinal	↓ H3K9ac; ↑ 5hmC/TET upon AMPK restoration	24–72 h (miRNAs earlier)
**miRNA remodeling**	Plasma/serum miR-181a/494 (oxidative), miR-378/486 (hypertrophic), miR-21/34a (fibro-oncogenic)	↑ miR-21/34a; rigidified profile	Human: acute + training periods	Shift back toward oxidative profile (miR-181a/494)	0–24 h
**Mitochondrial quality**	PGC-1α, MFN2, DRP1, mtDNA damage, LC3-II	↓ mitophagy; ↑ DRP1; ↑ mtROS	Robust experimental; emerging human data	↑ mitophagy; ↑ PGC-1α; reduced mtROS	24–72 h

Notes: PBMC, peripheral blood mononuclear cells; WB, Western blot; ELISA, enzyme-linked immunosorbent assay; ChIP, chromatin immunoprecipitation. The biomarker axes, predicted directions in overdrive, and suggested sampling windows are grounded in published evidence on AMPK–mTOR signaling/autophagy, NAD^+^ partitioning, redox control, and exercise-induced epigenetic/miRNA remodeling [1,3,6,10,17,19,30,38,61,62,66,68,69,73,74,75,76,77,78,80,82,110,111].

## Data Availability

No new data were generated or analyzed in this study. Data sharing is not applicable to this article.

## Abbreviations

AMPK	AMP-activated protein kinase
AOI	Adaptive Oscillation Index
BCAA	Branched-chain amino acids
CaMKKβ	Ca^2+^/calmodulin-dependent protein kinase kinase β
DNMT	DNA methyltransferase
EGCG	Epigallocatechin gallate
EPO	Erythropoietin
ER	Endoplasmic reticulum
FOXO	Forkhead box O transcription factors
GH	Growth hormone
GSH/GSSG	Reduced/oxidized glutathione
HAT	Histone acetyltransferase
HDAC	Histone deacetylase
HIF-1α	Hypoxia-inducible factor 1 alpha
HRV	Heart rate variability
IGF-1	Insulin-like growth factor 1
LC3	Microtubule-associated protein 1A/1B-light chain 3
LLM	Large language model
mTOR	Mechanistic target of rapamycin
mTORC1	Mechanistic target of rapamycin complex 1
mTORC2	Mechanistic target of rapamycin complex 2
NAD^+^	Nicotinamide adenine dinucleotide (oxidized form)
NAMPT	Nicotinamide phosphoribosyltransferase
NF-κB	Nuclear factor kappa B
NRF2	Nuclear factor erythroid 2–related factor 2
PARP	Poly(ADP-ribose) polymerase
PBMC	Peripheral blood mononuclear cells
PGC-1α	Peroxisome proliferator-activated receptor gamma coactivator 1 alpha
ROS	Reactive oxygen species
SAM	S-adenosylmethionine
SAH	S-adenosylhomocysteine
SIRT1	Sirtuin 1
S6K1	Ribosomal protein S6 kinase beta-1
TBARS	Thiobarbituric acid reactive substances
TET	Ten–eleven translocation dioxygenase
TFAM	Mitochondrial transcription factor A
ULK1	Unc-51-like autophagy activating kinase 1
UPR	Unfolded protein response
